# Learning Curve of Total Hip Arthroplasty in Direct Anterior Approach without Requiring Corrective Osteotomy for Hip Dysplasia

**DOI:** 10.1111/os.13231

**Published:** 2022-04-07

**Authors:** Kaiwei Shen, Eryou Feng, Feitai Lin, Yan Weng, Jinhua Chen

**Affiliations:** ^1^ Department of Arthrosis Surgery Fuzhou Second Hospital Affiliated to Xiamen University Fuzhou China; ^2^ Medical Department of Fujian Medicine University Union Hospital Fuzhou China

**Keywords:** Complications, Cumulative sum analysis, Direct anterior approach, Hip dysplasia, Learning curve

## Abstract

**Objective:**

To explore the learning curve of total hip arthroplasty in direct anterior approach (DA‐THA) without requiring corrective osteotomy for patients with unilateral developmental dysplasia of the hip (DDH) through the evaluation of clinical and radiographic results.

**Method:**

From December 2015 to January 2021, we retrospectively evaluated a surgeon's first 100 patients with unilateral hip dysplasia (Crowe I‐III) who underwent DA‐THA. All procedures were performed by a fellowship‐trained joint surgeon. Cementless hemispheric porous‐coated acetabular cups and tapered cementless stems were used in all hips. The radiographic data, including leg length, the height of the center of rotation, femoral head offset, the cup anteversion and inclination angle, were measured. The cumulative sum analysis (CUSUM) and risk‐adjusted cumulative sum analysis (RA‐CUSUM) were used to determine the learning curve of DA‐THA for each patient's operation time. By analyzing the operation time, complication rate, postoperative length of hospitalization and creatine kinase (before surgery and the third day after surgery), estimated blood loss, Harris score, radiographic data were compared between the different stages of the learning curve.

**Results:**

The mean follow‐up time was 35.45 ± 16.82 months. The CUSUM method obtained the maximum turning point of the curve at 43 cases, which divided the learning curve into Learning Period and Mastery Period. The CUSUM learning curve was best modeled as a cubic curve with the equation: CUSUM (min) = 0.001*x* ^3^ − 0.495x^2^ + 33.60*x* − 10.00, which had a higher *R* ^2^ value of 0.967. The pre‐operative data, creatine kinase, estimated blood loss and postoperative Harris scores of the two stages were not statistically significant (*P* > 0.05). The mean operation time was 118 min in the Learning Period and 87 min in the Mastery Period. Statistically significant differences were detected in the operation time (*P* < 0.001), postoperative length of hospitalization(*P* = 0.024), and postoperative leg length discrepancy (*P* = 0.012) between the two stages. The overall complication rates were 27.9% in the Learning Period and 12.3% in the Mastery Period (*p* = 0.049). The overall outliers of radiographic data were 34 cases in the Learning Period and 31 cases in the Mastery Period (79.07% vs 54.39%, *P* = 0.010).

**Conclusions:**

The DA‐THA is a valuable alternative to achieve satisfactory clinical results for mild‐to‐moderate DDH patients. Furthermore, accurate analysis of the learning curve of DA‐THA for hip dysplasia by the CUSUM method showed that the surgeons need to finish about 43 cases to master the technique.

## Graphical abstract

We used the cumulative sum analysis to obtain the learning curve of first 100 patients with unilateral hip dysplasia who underwent total hip arthroplasty in direct anterior approach without requiring corrective osteotomy. Surgeons need to finish about 43 cases to master the technique in terms of operative time, complication rate, and radiographic data.

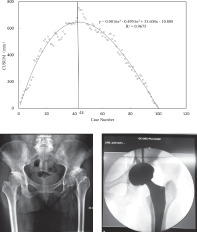

## Introduction

It is reported that developmental dysplasia of the hip (DDH) represents 2.6% to 9.1% of total cases of total hip arthroplasty (THA) and is the main cause of THA in young people—about 21% to 29%^1^, ^2^. In China, there were about 16.05 million DDH patients with a total prevalence rate of 2.245%, most of which are less severe hip dysplasia^3^. In comparison to those in THA with primary osteoarthritis, DDH patients are not only at a higher risk for postoperative complications due to the altered anatomy^4^ but also place higher demands on the function of the hip joint^5^. The direct anterior approach (DA‐THA) has been reported to yield optimized mid‐and‐long term clinical and radiographic outcomes for hip dysplasia and it improves the postoperative satisfaction of young patients.^6^ As the typical representative of enhanced recovery after surgery (ERAS) in joint surgery cases,^7^ DA‐THA is a minimally invasive surgical procedure that enables the hip muscles to be accessed through inter‐nerve and inter‐muscle pathways, providing the advantage of intraoperative fluoroscopy, lower risk of dislocation^8^, and less muscle damage^9^. Hence, at least theoretically, a direct anterior approach seems to be the most appropriate approach of THA for hip dysplasia. However, the potential needs for technique and auxiliary equipment of DA‐THA are much higher than that of other THA approaches^10^ and it is generally recommended for simple and primary hip disorders, such as Crowe type I‐II DDH^11^, where DA‐THA is optimal to use a regular monobloc prosthesis..^12^. Early selection of appropriate cases is a key element for surgeons to avoid high complications^13^ and master the challenging technique^14^. Therefore, it is important to take surgical learning curves into account when interpreting outcome data that is acquired during an implementation period. This may especially be the case for a technically challenging procedure like DA‐THA.^15^, ^16^, ^17^

Although the learning curves for some major DA‐THA procedures have been well established^10^, ^15^, ^17^, ^18^, there is room for improvement in the reliability of statistical methods. To the best of our knowledge, the cumulative sum analysis (CUSUM) and risk‐adjusted cumulative sum analysis (RA‐CUSUM), which indicates when a state has reached a steady level of performance and determines when proficiency is achieved^19^, ^20^, have been evaluated in medical procedures^21^, but rarely in the DA‐THA. In addition, we found that patients with high migration hips presented higher demand in surgical skills due to osteotomy techniques^22^, potentially leading to heterogeneity in the assessment of the learning curve. Therefore, the purpose of this study is to assess the learning curve using the CUSUM and RA‐CUSUM methods by a single surgeon adopting DA‐THA without requiring corrective osteotomy in unilateral DDH patients and to compare clinical outcomes according to this learning curve.

The aim of the present study was: (i) to determine a minimum number of cases required for a single surgeon to master DA‐THA in the first 100 DDH patients; (ii) to evaluate the complication rates, clinical and radiographic results at different periods of the learning curve; and (iii) to prove the effectiveness of DA‐THA in the treatment of DDH.

## Patients and Methods

### Inclusion and Exclusion Criteria

The inclusion criteria were: (i) adult patients with unilateral DDH; (ii) the first 100 patients receiving cementless DA‐THA without corrective osteotomy from a single surgeon in our institution between December 2015 and January 2021; (iii) patients who were able to provide information during postoperative follow‐ups; and (iv) retrospective study.

The exclusion criteria were: (i) patients with severe hip dysplasia who required corrective osteotomy (e.g., partial Crowe type III, type IV, or high dislocation of Hartofilakidis classification); (ii)patients with bilateral hip dysplasia; (iii) patients with a body mass index (BMI) > 35^23^; and (iv) patients who were missing hospital data.

The uniform selection of unilateral DDH patients without osteotomy can help reduce the bias of the assessment of the learning curve due to individual differences. It is widely known that complex osteotomy for patients with high migration hips can result in increased injury and operation time. Similarly, the affected side was chosen rather than both sides. This eliminated bias while also increasing the accuracy of the design results by referring to the normal side.

### Patients

Institutional Review Board approval was obtained for the study. The first 100 unilateral DDH patients with contralateral normal hip by DA‐THA were included retrospectively from December 2015 to January 2021 at the Joint Surgery Center of Fuzhou Second Hospital in China where over 2000 cases of DA‐THA were performed by the submission date. The patients were followed for 35.45 ± 16.82 months and examined at the clinic after one, three, and six months postoperatively to identify any complications and functional outcomes. Operative time, post‐operative length of hospitalization (post‐op LOH), estimated blood loss (EBL), creatine kinase (before surgery and 3 days after surgery), preoperative and final follow‐up Harris score, and perioperative complications were documented for each patient.

Surgical complications were defined as periprosthetic fracture, unacceptable LLD, readmission for postoperative pain or incision‐related complications, dislocation, and prosthetic joint infection. Periprosthetic fractures are fractures caused by incorrect intraoperative operations and include fractures that are immediately detected by intraoperative fluoroscopy and occultation fractures discovered during postoperative follow‐up. The complications of readmission were defined as patients with postoperative pain severely affecting their lives, patients with a VAS score >8, and those who still failed after oral analgesic therapy. Similarly, patients with poor postoperative incision healing or superficial infection that required further treatment as complications of readmission were included. The classification of other complications is the same as those described by Woolson *et al*.^24^.

### Surgical Technique

All procedures were performed by a fellowship‐trained joint surgeon with extensive posterior lateral hip arthroplasty and revision capabilities before experiencing DA‐THA. The surgeon was not exposed to DA‐THA during the residency or attending stages. During this period, he attended a 1‐year training course at another hospital, where he consulted several other surgeons with an abundance of experience in the DA approach, conducted surgical observations, and completed cadaver courses.

#### Anesthesia and Position

Each patient was positioned in a supine pose on a fracture table (Hana Table, Union City, CA) under general anesthesia and femoral nerve and sciatic nerve block anesthesia. The pubic symphysis of each patient was positioned directly at the folding mark of the table.

#### Approach and Exposure

A direct anterior approach was used for all patients with an incision of approximately 10 cm in length, from the anterior superior iliac spine, distally pointing to the fibula head. The Hueter interval between the tensor fascia lata (TFL) and sartorius was obtained, and the fascia lata was cut lengthways, ~2 cm from the anterior edge of the TFL, to avoid injuring the lateral femoral cutaneous nerve. A blunt anatomical separation was performed along the medial side of the TFL into the deep layer. The surgeon coagulated the ascending branches of the lateral femoral circumflex artery and made an “inverted T” incision in front of the joint capsule. The assistant placed the hip in inclination and pronation to assist the surgeon in releasing the lateral portion of the capsule, and in extorsion to facilitate the exposure of the medial and lower capsule. The femoral head was resected with a reciprocating saw, based on the distance from the lesser trochanter using the template design for attaining the proximal stability of the sleeve.

DA‐THA provides greater exposure to the acetabulum than other approaches. The acetabular retractor was placed to slightly flex the hip and help expose the acetabulum and release the reflex head of the femoral rectus. Three Homman retractors were placed in the 4, 8, and 11 o'clock positions on the posterolateral, anterior acetabulum, and medial capsular incision. Attention was paid to avoid the compression of retractors on the TFL.

A typical femoral release can only begin when adequate exposure of the proximal femur is achieved by lateralizing and elevating manipulations when the hip is overextended, adducted, and externally rotated. This procedure is performed by placing sheets beneath the pelvis preoperatively or by using a fracture table.

#### Fixation or Placement of Prosthesis

Cementless hemispheric porous‐coated acetabular cups and tapered cementless stems were used in the hips of all patients. The acetabulum was reconstructed at its anatomical rotation center, which can be found by following the transverse and round ligaments, and the acetabulum was reamed to the appropriate size and placed in an appropriate cup and liner. The femoral anteversion was then determined by the transepicondylar line of the femoral condyle as the reference and the combined anteversion of the limb was set under 55°, due to substantial anteversion of the acetabulum and femur in DDH patients. A standard proximal broaching and distal reaming process was then performed. More elevation and lateral shifting of the proximal femur, and peeling‐off of the TFL attachment and partial release of the piriformis, were required by patients with tissue contractures or extensive coverage of the cotyloid fossa. The femoral prosthesis was then inserted into the medullary cavity and reduced as required. It was confirmed that the prosthesis and screw were in place at the upper margin of the obturator foramen by intraoperative C‐arm X‐ray. Finally, the wounds were flushed and sutured.

#### Postoperative Reconstruction

A standard program of multimodal analgesia, physiotherapy, and enhanced recovery was launched immediately following the conclusion of surgery. Antibiotics were administered *via* intravenous injection during the first 24 hours and the oral administration of rivaroxaban was recommended for 5 weeks postoperatively. Patients were instructed to wear and take off socks on the first day and encouraged to walk using crutches as soon as possible based on their conditions.

### Radiograph Data

An anteroposterior pelvic X‐ray was obtained for each patient at the final outpatient follow‐up, and the Star‐PACS imaging system was used for this (YiLianZhong, Xiamen, China). The measurements were examined using digital imaging analysis software (Materialise interactive medical image control system, ©2014 Materialise, Leuven, Belgium).

So that bias could be avoided, the order of measurements was assigned at random to two orthopedic surgery residents who had no access to the information of the patients. The residents evaluated each radiograph and the means of the two values were used for study measurements. To account for magnification, the radiographic distance was determined using digital imaging analysis software corresponding to the standard ruler of the actual length provided by the anteroposterior pelvic X‐ray. The true target length was obtained by dividing the true size into the measured size of the ruler and multiplying it by our measured value.

### Measurement Details

#### Leg Length Discrepancy

Leg length discrepancy (LLD) was determined in millimeters, by measuring the difference between the acetabular teardrops line and a bilateral line between the lesser trochanters. The difference between the operated and non‐operative sides was then compared, and LLD > 10 mm was recorded as unacceptable^22^, ^25^.

#### Femoral Head Offset

To determine the femoral head offset, a line was drawn parallel to the femoral shaft. The distance from that line to the center of the femoral head was measured, and the difference between the operated and non‐operative sides was compared. A femoral head offset discrepancy >5 mm was recorded as an outlier^25^.

#### Hip Center of Rotation

In this study, the hip center of rotation (COR) is the vertical and horizontal distances from the center of the femoral head to the teardrops. The difference between the operated and non‐operative side radiographic vertical and horizontal distance was calculated to determine any change in hip COR. Vertical or horizontal distance discrepancies >5 mm were recorded as outliers^26^.

#### Cup Anteversion and Inclination

The acetabular cup anteversion angle was measured using the Lewinnek method: the formula for version = ARC sine (minor axis)/ (major axis), and an anteversion angle >25° or <5° was recorded as an outlier^27^. To measure the acetabular cup inclination angle, an angle was drawn between the cup long axis and the acetabular teardrops, and an inclination angle >50° or <30° was recorded as an outlier^28^.

### Statistical Analysis

#### Cumulative Sum Analysis

The CUSUM technique is a time‐weighted control chart method used for identifying inflection points. It is not discernible in other approaches and calculates the sequential difference between raw data and the mean value^20^, ^21^. In this study, the chronological order of cases was taken as the X‐axis, and the CUSUM based on the average operation as was taken as the Y‐axis, to plot and fit the learning curve. An upward slope indicated an increasing trend of operative time, and a downward slope indicated a decreasing trend, in comparison to the mean value. The curve fitting was deemed to be successful when *p* < 0.05, and the goodness of fit was judged by *R* ^2^. Different learning curve stages were divided according to the vertex of the CUSUM fitting curve, which was taken as the minimum cumulative number of surgical cases required to cross the learning curve.

#### Risk‐Adjusted Cumulative Sum Analysis

Risk‐adjusted cumulative sum analysis (RA‐CUSUM) helps explain the difference between predicted and actual events and is a further extension of the CUSUM method^21^. The surgical complications that are defined in this study and the outliers of radiographic data were selected for assessing the failure of DA‐THA. Multivariate analysis was used for evaluating each risk factor that is associated with DA‐THA failure, and the data was considered for logistic regression to calculate surgical failure probability. Finally, each included case was plotted from left to right on the horizontal axis, the RA‐CUSUM curve shifting downward representing DA‐THA success and upward representing failure.

### Statistical Analysis

All statistical analyses were performed using SPSS Statistics, (v.22.0 for Windows; SPSS Inc., Chicago, IL, USA). Continuous variables were expressed as mean value ± standard deviation, while categorical variables were expressed as percentages. The *t*‐test, Fisher's exact test, chi‐square test, and rank‐sum test were all performed as a means of examining whether demographics and clinical data differed significantly between the two groups divided by the learning curve. *P* < 0.05 was considered to be a significant difference.

### Results

#### Learning Curve Results

**Fig. 1:**
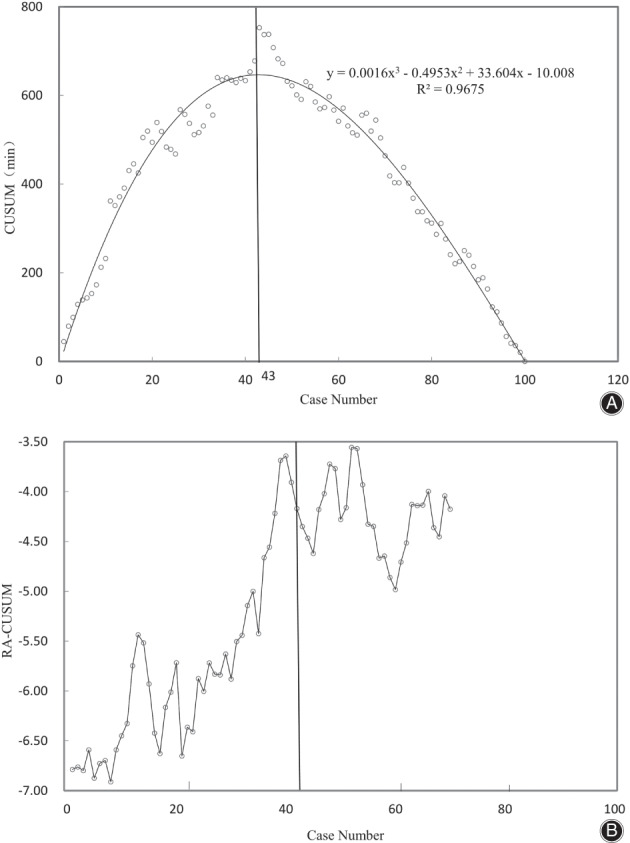
The maximum turning point of the learning curve at the 43rd case and 40th case in the CUSUM and RA‐CUSUM method, respectively

The CUSUM learning curve was best modeled as a cubic curve using the equation: CUSUM (min) = 0.001*x* ^3^ − 0.495*x* ^2^ + 33.60*x* − 10.00 (*X* was the number of surgical cases), with a higher *R* ^2^ value of 0.967 (Fig. 1A). The fitting curve reached the top at the 43rd case and the RA‐CUSUM method was used to obtain the maximum turning point of the curve at the 40th case (Fig. 1B). The collective curve trends and results from the CUSUM and RA‐CUSUM method determined the cutting point of the 43rd case, dividing the learning curve into the Learning Period and the Mastery Period.

#### Demographic Results

Twenty‐eight males and 72 females were included in the study. Preoperative Hartofilakidis classification included the dysplasia of 79 cases and low dislocation of 21 cases. A total of 100 Crowe type I, II, and III hips consisted of 80 type‐I hips, 14 type‐II hips, and six type‐III hips. No significant differences were observed between the two groups in terms of demographic data (*P* < 0.05), Crowe classification (*P* = 0.313), Hartofilakidis classification (*P* = 0.964), or pre‐operative Harris score (*P* = 0.630, Table 1).

**TABLE 1:** Pre‐operative data

Group	Age (years)	Gender (M/F)	BMI (kg/m^2^)	Laterality (R/L)	Pre‐op Harris score	Classification					
						Crowe			Hartofilakidis		
						I	II	III	D	L	H
Learning period	55.65 ± 11.39	14/29	27.79 ± 4.26	22/21	54.56 ± 10.70	36	7	0	34	9	0
Mastery period	60.37 ± 14.05	14/43	28.11 ± 3.94	29/28	55.67 ± 11.64	44	7	6	45	12	0
*t*/*χ* ^2^/*z*	−1.782	0.777	−0.366	0.001	−0.483	1.010			−0.045		
*p* Value	0.078	0.378	0.716	0.977	0.630	0.313			0.964		

M, male; F, female; BMI, body mass index; R, right; L, left; D, dysplasia; L, low dislocation; H, high dislocation.

#### Clinical Results

As can be seen in Table 2, the Learning Period demonstrated an increase in operating time (117.79 ± 34.95 *vs* 87.09 ± 22.32, *P* < 0.001), post‐op LOH (5.98 ± 3.39*vs* 4.02 ± 1.41, *P* = 0.024),and post‐op LLD (6.11 ± 4.63 *vs* 4.19 ± 2.72, *Pp* = 0.012), all of which were statistically significant. No significant difference was evident between the two stages in EBL (*P* = 0.121), postoperative Harris score(*p* = 0.876), and creatine kinase(*P* = 0.601).

**TABLE 2:** Post‐operative data

Group	Operation time (min)	Post‐op LOH (days)	Post‐op LLD (mm)	Creatine kinase (D3–0) (U/L)	EBL (mL)	Post‐op Harris score
Learning period	117.79 ± 34.95	5.98 ± 3.39	6.11 ± 4.63	661.40 ± 422.11	553.02 ± 339.95	86.01 ± 4.92
Mastery period	87.09 ± 22.32	4.02 ± 1.41	4.19 ± 2.72	619.53 ± 366.89	460.88 ± 242.63	86.87 ± 5.81
*t*	5.290	2105	2.030	0.524	1.565	−0.467
*p* Value	0.000*	0.024*	0.012*	0.601	0.121	0.876

LOH, length of hospitalization; post‐op, post‐operative; EBL, estimated blood loss; LLD, leg length discrepancy; D3–0 the difference of creatine kinase between the third day after surgery and before surgery.

* Significant difference.

#### Complications and Treatment

The overall complication rates were 27.9% (12 out of 43) in the Learning Period and 12.3% (seven out of 57) in the Mastery Period (*P* = 0.049, Table 3). The complications in the learning period included one case of anterior dislocation, one of readmission for poor postoperative wound healing (dressing being changed until the wound healed), and three of intraoperative greater trochanter fracture (fracture fixation with wires). One female patient experienced anterior dislocation of the hip while urinating in the bed 2 days following the operation. The radiograph showed satisfactory recovery of the hip rotation center, with a cup inclination angle of 66° and a combined anteversion angle of 28°. No further dislocation was recorded following manual reduction until the final follow‐up. It is believed that poor posture and excessive cup inclination angle are the main mechanisms that lead to dislocation. The specific complication related to the Mastery Period included one case of periprosthetic staphylococcus aureus joint infection 1 month following the operation The patient was asked to perform one‐stage revision arthroplasty with debridement, and given antibiotics. At the time of writing, the 15‐month postoperative radiographs showed well‐fixed implants without any sign of loosening or interval change in alignment, and the patient has been pain‐free.

**TABLE 3:** Complications

Group	Complications						
	Unacceptable LLD (>10 mm)	Postoperative pain	Poor wound healing	Periprosthetic fractures	Dislocation	Infection	Total
Learning period	6 (14.0%)	1 (2.3%)	1 (2.3%)	3 (7.0%)	1 (2.3%)	0	12 (27.9%)
Mastery period	3 (5.3%)	1 (1.8%)	0	2 (3.5%)	0	1 (1.8%)	7 (12.3%)
t/χ^2^	1.323	0.000	‐	0.111	‐	‐	3.893
*p* Value	0.250	1.000	0.430	0.746	0.430	1.000	0.049*

*P* value means the overall complication rates of radiographic data, LLD leg length discrepancy.

* Significant difference.

#### Radiographic Results

Regarding the radiographic data, the reliability of both intrarater and interrater values were interpreted as excellent (≥0.75) for every parameter that was evaluated, with ICC values of 0.809–0.965^29^. Although no significant differences in cup inclination angle (41.89 ± 6.68 vs. 41.95 ± 5.38, *P* = 0.962), cup anteversion angle (17.89 ± 6.00 *vs*. 17.04 ± 5.08, *pP* = 0.495), and femoral offset change (6.37 ± 4.01 *vs*. 5.24 ± 3.56, *P* = 0.147) were observed in the two stages, the Mastery Period had a significantly lower horizontal and vertical change of hip COR than the Learning Period (3.54 ± 2.41 *vs*. 5.34 ± 4.52, *P* = 0.013; 2.90 ± 2.02 *vs*. 4.31 ± 3.14, *P* = 0.008). As can be seen in Table 4, the overall outliers of radiographic data were 31 cases in the Mastery Period and 34 cases in the Learning Period (54.39% *vs*. 79.07%, *P* = 0.010).

**TABLE 4:** Comparison and outlier of patient radiographic data between learning period and mastery period

Radiographic Variables	Learning period	Mastery period	t/χ^2^	*P* Value
Post‐op cup inclination angle (°)	41.89 ± 6.68	41.95 ± 5.38	−0.996	0.962
Post‐op cup anteversion angle (°)	17.89 ± 6.00	17.04 ± 5.08	0.759	0.495
Post‐op femoral offset change (mm)	6.37 ± 4.01	5.24 ± 3.56	0.658	0.147
Post‐op horizontal change (mm)	5.34 ± 4.52	3.54 ± 2.41	3.614	0.013*
Post‐op vertical change (mm)	4.31 ± 3.14	2.90 ± 2.02	2.210	0.008*
No. of outlier of LLD (>10 mm)	6	3	2.260	0.133
No. of outlier of cup inclination (>50° or < 30°)	6	1	3.886	0.049*
No. of outlier of cup anteversion (>25°or <5°)	7	1	5.191	0.023*
No. of outlier of femoral offset change (>5 mm)	24	20	4.273	0.039*
No. of outlier of horizontal change (>5 mm)	13	7	4.937	0.026*
No. of outlier of vertical change (>5 mm)	9	6	2.081	0.149
Total	34	31	6.564	0.010*

Post‐op is post‐operative, LLD leg length discrepancy; Post‐op change means the difference between the operated and non‐operative side on anteroposterior pelvic X‐ray at the last outpatient follow‐up.

* Significant difference.

## Case Presentation

**Fig. 2:**
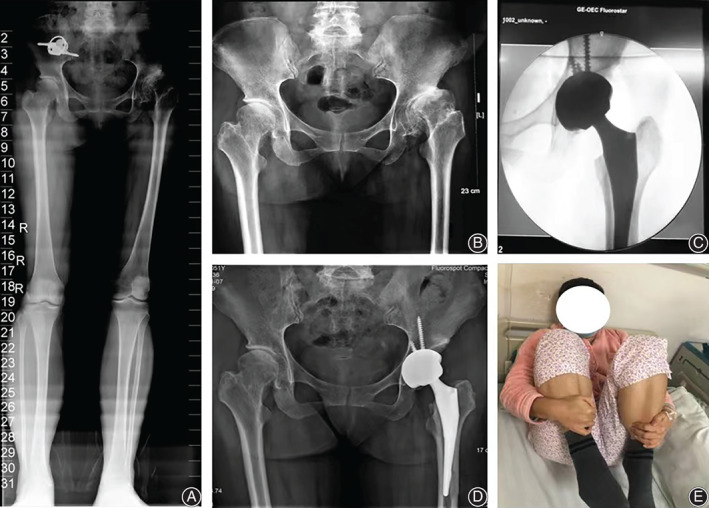
The 73rd case on the learning curve with unilateral hip dysplasia treated with DA‐THA. (A) Preoperative full‐length radiograph of lower extremity. (B) Preoperative anteroposterior radiograph of pelvis. (C) Intraoperative radiograph. (D) Postoperative anteroposterior radiograph of pelvis in the last follow‐up for 13 months. (E)The hip activity on the first postoperative day

A 51‐year‐old woman, with a body mass index of 26.8 kg/m^2^, who complained of chronic progressive left hip pain and who had limped for over 12 years was admitted into our clinic in December 2019. An anteroposterior (AP) radiograph of the pelvis and full‐length radiograph of the lower extremities found Crowe type II DDH of the left hip with subluxation and a leg length discrepancy of 20 mm (Fig. 2A, B).

This was the 73rd DA‐THA case in the learning curve. A cementless porous‐coated acetabular cup of 52 mm with a tapered cementless stem of 7.5‐size (Zimmer, Warsaw, IN, USA) was required, with three acetabular screws for fixation. The duration of the operation was 80 min and the estimated blood loss was 450 mL. Intraoperative radiographs demonstrated a well‐aligned total hip prosthesis (Fig. 2C). Hip activity showed improvement on the first postoperative day (Fig. 2E). According to the pelvis radiograph at the final follow‐up after 13 months, the inclination angle of the acetabular shell was 48° and the anteversion was 19°, which were in the Lewinnek safe zone. The anatomic leg length discrepancy was <10 mm, and the deviation of the hip rotation center was <5 mm (Fig. 2D).The Harris score had improved from 64 points before the operation to 86 points at the final follow‐up. No complications were found, including dislocation, infection, wound issue, fracture, nerve palsy, and moderate to severe limping.

## Discussion

Recently, several studies have documented the individual learning curve of surgeons when using DA‐THA. A report analyzed13 surgeons performing 4138 procedures through the direct anterior approach for primary osteoarthritis over a 4‐year period. The results suggest that 50 or more procedures need to be performed by a surgeon before the rate of revision is no different from performing 100 or more procedures^18^. Another clinical research project evaluated the first 500 consecutive DA‐THAs by a single surgeon. It was found that the incidence of major complications in patients decreased with increasing experience, with the most dramatic improvement after the first group of 100 cases^10^. Kong *et al*. reported a learning curve of first‐100 cases with unilateral DA‐THA through Cumulative Summation analysis, indicating that complication rates and operating time reached acceptable and steady state after 88 cases and 72 cases respectively^15^. It is not difficult to see that proficiency in the DA‐THA, as with many surgical techniques, is achievable. Even so, due to differences in statistical design, inclusion criteria, evaluation criteria and outcome measures, the learning curve of the minimum number of cases to complete this procedure is still controversial. Therefore, we designed this study in the hope of contributing to an accurate assessment of the DA‐THA learning curve.

### A Minimum Number of Cases to Complete a Learning Curve for DA‐THA

When determining the learning curve, we considered not only the operative time but also the failure rate of DA‐THA because total hip arthroplasty completion needed technical mastery to achieve surgical outcomes, including lower complication rate and optimal implant position. In the present study, the two phases of the learning curve were defined as the Learning Period and the Mastery Period. The RA‐CUSUM method was applied to evaluate the parameters affecting surgical outcomes. The minimum surgical failure was observed by the 40th case, as seen in Fig. 1B, and the 40th case was located before the plateau (the 43rd case), as seen in Fig. 1A. This indicates that although the learning curve had been overcome by the 40th case in terms of DA‐THA failure, the probability of operative time did not reach the lowest point until case 43. Therefore, case 43 was regarded as the point at which to achieve competence in DA‐THA in this study.

### Evaluation of the Clinical Results in the Learning Curve

CUSUM analysis showed that a steady state of operating time was reached at around 87 min for the Mastery Period, which is similar to previous reports^15^, ^16^, ^30^. This improvement of the operating time may be attributed to a reduction in fluoroscopy time and an enhanced ability in assistance, especially the precise hyperextension, adduction and external rotation of the leg, which help expose the femur efficiently. With the consideration of the length of hospitalization (LOH), which was affected by many issues in our department including treatment of comorbidity, length of preoperative examinations and the pressures on beds, the post‐op LOH we used, indicated that the rapid subsequent recovery in the Mastery Period of DA‐THA was improved. A large comparative study found patients with DDH achieved comparable early functional results compared to patients with osteoarthritis (OA)^31^. However, compared with other literatures^24^, ^32^, our patients were in the hospital longer, which may be attributed to the higher requirement by doctors for enhanced recovery pathways, including wearing and removing of socks independently, and early gait improvement. Although the DA‐THA is considered to be a minimally‐invasive approach, a recent study for DDH clarified that more muscle damage was produced through the DAA^33^. Creatine kinase measurements have been proven to be reliable when assessing muscle damage, and usually reach their peak on days 2 and 3 postoperatively^15^, ^34^. In our study, a trend towards lower serum levels was noticed in the Mastery Period, indicating that a reduction in muscle damage was associated with improved dissection technique. However, we found no significant results between periods of the learning curve, which might be attributed to the standardized operation of DA‐THA and relatively small sample size. Similarly, estimated blood loss decreased but not by a significant amount between periods of the learning curve. The result was similar to other reports with an average blood loss of 526 mL by fellowship‐trained arthroplasty specialists,^35^ which was thought to be due either to technical difficulties in femoral preparation or a steep learning curve. The confounding factors that were ignored in our study, including the pre‐operative level of hemoglobin and medical comorbidities, may be part of the reason why we noticed no statistical difference in EBL. Hence, it may be necessary to control confounding factors to detect a statistically significant difference.

### Evaluation of the Complication Rate in the Learning Curve

In the current study, the RA‐CUSUM method indicated that the incidence of complications remained stable by the 40th case. The overall complication rate was 19%, and we found a decrease in the Mastery Period of 12.3% as compared to the Learning Period of 27.9%, which were statistically significant. The results were similar to a study by Foissey *et al*.^16^, which reported significant differences in the learning curves of both senior and trainee surgeons using DA‐THA. However, due to the different definitions of complications, Kong *et al*.^15^ reported higher rates of complications of 16% to 44%. Woolson *et al*.^24^ reported a 9% incidence of major complications in a group of community practice orthopedists in their learning curve with the DA‐THA. By the criteria used in Woolson's study, our complication rate was similar at 7%.

### Variation of the Femoral Fracture Rate in the Learning Curve

Our femoral fracture rate with the DA‐THA was 5.0%, including two unfixed chip (small fragmented) fractures, and three fixed fractures involving repair of almost the entire greater trochanter with wires. We believe that this incidence of fracture in our study was related to the surgical techniques. First, a fracture might occur when the retractor was inserted into the tip of the greater trochanter and retraction forced without adequate soft‐tissue release. Second, without the proper use of special surgical tables, a bone hook would be used for the proximal femur elevation, putting the tip of the greater trochanter at risk of chip fracture. Besides excessive anteroflexion, femoral stems in patients with DDH were at risk of being undersized, or of being placed in malalignment or malrotation^13^, possibly increasing the risk of femoral fracture. The result was similar to several studies, reporting this complication with a rate varying between 1% and 6.5% during the learning curve of DAA^24^. However, unlike a decrease in periprosthetic fracture rates reported by Hartford from the 1st 100 cases to the last 100 cases^10^, our femoral fracture rate was not statistically correlated with the learning curve. Foissey *et al*. also noticed that there was not a decrease in greater trochanter fractures with experience^16^. Elderly, female, and osteoporotic patients were associated with an increased risk of periprosthetic femoral fractures^36^. We believe that the learning curve may not be the only factor affecting intraoperative fracture rate. Thus, for beginners, take time when learning this technique especially on femoral exposure, and pay attention to defined exclusion criteria in DA‐THA.

### Variation of the Leg Length Discrepancy in the Learning Curve

Although we did not include patients who had undergone osteotomy, LLD is a common complication and concerns related to LLD can cause anxiety and depression in DDH patients^37^. In agreement with the results published by Woolson, accurate leg length within 10 mm was achieved in 91% of our cases. With the use of fluoroscopy assistance in all of the cases, the incidence in perceived leg length discrepancies declined from 6% in the Learning Period to 3% in the Mastery Period. We recommend surgeons utilize the benefit of fluoroscopy and supine positioning to reduce the likelihood of complications from LLD.

### Evaluation of the Radiographic Results in the Learning Curve

Implant positioning is important for optimum hip stability, avoiding early loosening and decreasing both bearing surface wear and revision rate. We noticed a significant improvement in the overall outliers of radiographic data from the Learning Period to the Mastery Period. At the beginning of the experience, there was a more lateral and cranial deviation in comparison with the COR in the healthy hip on the contralateral side. Difficult exposure during the Learning Period induced a poor visualization of anatomical landmarks for DDH patients of a small, shallow true acetabulum. Out of fear of not being able to maximize cup coverage for improving cup stability, the surgeons tended to overreem the anterior or posterior acetabular column. After an initial adjustment period, based on the analysis of post‐operative radiographs, the surgeons corrected their motion by reaming the acetabulum postero‐superiorly and achieved a more accurate reconstruction of the horizontal and vertical COR. The supine position in DAA creates less alteration of the pelvic orientation and allows intraoperative fluoroscopy, avoiding the important mistakes of cup positioning, which for us may be the reason why there was no significant difference with the post‐op femoral offset change, inclination angle and anteversion angle.

### Advantages of DA‐THA

As a superficial surgical approach, DA‐THA can well expose the acetabulum, helping surgeons with direct visualization and manual palpation to verify anatomic cup positioning. The position of the pelvis is fixed in the supine position, where the acetabular component sizing and positioning would be not affected by the change of the pelvis position caused by the pull of the retractor in the lateral position. What is more, the potential advantage of DA‐THA is to easily obtain the superiority of the optimum component position, impingement‐free motion, and stability of the hip through the intraoperative use offluoroscopy.^38^

Although traditional (lateral, posterolateral, and posterior) THA approaches have been used with excellent results^39^, they damage periarticular muscles which are already weak for DDH patients, with postoperative dislocation rates of up to 16.6%^13^. Invasion of the short externals, although properly repaired, might increase the risk of instability and dislocation^40^. In addition, interruption of the branch of the femoral artery impairs osteointegration on the host bone‐prosthesis interface and increases the risk of nonunion at the osteotomy site^41^. In contrast, dislocation rates are 22%^42^ lower when using the DAA compared to the posterior approach, which is attributed to abductor muscle preservation and less soft‐tissue damage, which also enable full weight‐bearing by 1 week postoperatively, compared with 3–16 weeks with traditional approaches^43^. Given these points, the DAA seems to be advantageous for DDH.

### Limitations of the Study

There are several limitations to this study. To provide a more accurate reference, we excluded dislocated dysplasia hips requiring corrective osteotomy, which facilitated the establishment of the learning curve. However, this also resulted in the inability to represent severe DDH patients in the technical difficulties of primary DA‐THA surgery. Similarly, we excluded patients with bilateral DDH, either 1‐ or 2‐stage DA‐THA, which has been widely recognized in previous learning curve clinical studies^10^, ^15^. The reason is that we need to control the operative time to ensure the accuracy of the learning curve, and the radiograph data of the operative sides are more persuasive based on the contralateral anatomical standards. Our results support that those surgeons who perform primarily joint arthroplasty need to finish about 43 cases to master the technique, but the result of a single experienced surgeon may not be reproducible for those who include arthroplasty as only a portion of their practice, and additional investigation is warranted.

When examining the complications analysis of the study, there are a few limitations as well. First, functional LLD after THA surgery is caused by scoliosis or pelvic obliquity^44^, and cannot be accurately measured by the perpendicular distance from the lesser trochanter to the inter‐teardrop line. Second, the frequency of lateral femoral cutaneous nerve injury or paresthesias was not routinely recorded in the perioperative period, but no obvious nerve symptoms were found at the last follow‐up, which may be due to the absence of osteotomy and the resolution of nerve injury symptoms in most cases within 6 months^45^. Third, although the assessment of cup anteversion is more accurate on a CT scan, this study was performed on X‐rays because CT data was lost for some patients. To compensate for the drawback, our department X‐ray technicians are specialized in the lower limb, and their images were assessed to minimize error.

### Conclusions

Based on this study, the learning curve was associated with decreased operative time, better clinical and radiographic outcomes. Conservatively, to attain technical competence in the treatment of DDH, a minimum of 43 cases is required for arthroplasty surgeons with a certain degree of DA‐THA experience. We recommend that surgeons learn and transition to the DA‐THA if the potential benefits of it outweigh the complication risks for their patients during the learning curve. In addition to attending cadaver courses and visitations, surgeons who decide to take on the challenge of the learning curve should pay more attention to fluoroscopy, surgical techniques and preoperative selection of patients.

## Data Availablility

The datasets used and/or analyzed during the current study are available from the corresponding author on reasonable request; please contact the corresponding author, Dr. Feng. Administrative permission was received from Fuzhou Second Hospital affiliated to Xiamen University (No. 47, Shangteng Road, Cangshan District, Fuzhou, China) to access the medical records.
